# Acquisition of Anoikis Resistance Up-Regulates Syndecan-4 Expression in Endothelial Cells

**DOI:** 10.1371/journal.pone.0116001

**Published:** 2014-12-30

**Authors:** Bruna Ribeiro Carneiro, Paulo Castanho A. Pernambuco Filho, Ana Paula de Sousa Mesquita, Douglas Santos da Silva, Maria Aparecida S. Pinhal, Helena B. Nader, Carla Cristina Lopes

**Affiliations:** 1 Departamento de Ciências Biológicas, Universidade Federal de São Paulo, Diadema, SP, Brazil; 2 Departamento de Bioquímica, Universidade Federal de São Paulo, São Paulo, SP, Brazil; University of Patras, Greece

## Abstract

Anoikis is a programmed cell death induced upon cell detachment from extracellular matrix, behaving as a critical mechanism in preventing adherent-independent cell growth and attachment to an inappropriate matrix, thus avoiding colonization of distant organs. Cell adhesion plays an important role in neoplastic transformation. Tumors produce several molecules that facilitate their proliferation, invasion and maintenance, especially proteoglycans. The syndecan-4, a heparan sulfate proteoglycan, can act as a co-receptor of growth factors and proteins of the extracellular matrix by increasing the affinity of adhesion molecules to their specific receptors. It participates together with integrins in cell adhesion at focal contacts connecting the extracellular matrix to the cytoskeleton. Changes in the expression of syndecan-4 have been observed in tumor cells, indicating its involvement in cancer. This study investigates the role of syndecan-4 in the process of anoikis and cell transformation. Endothelial cells were submitted to sequential cycles of forced anchorage impediment and distinct lineages were obtained. Anoikis-resistant endothelial cells display morphological alterations, high rate of proliferation, poor adhesion to fibronectin, laminin and collagen IV and deregulation of the cell cycle, becoming less serum-dependent. Furthermore, anoikis-resistant cell lines display a high invasive potential and a low rate of apoptosis. This is accompanied by an increase in the levels of heparan sulfate and chondroitin sulfate as well as by changes in the expression of syndecan-4 and heparanase. These results indicate that syndecan-4 plays a important role in acquisition of anoikis resistance and that the conferral of anoikis resistance may suffice to transform endothelial cells.

## Introduction

The extracellular matrix (ECM) affects many aspects of cell behavior, including the migratory properties of cells, their morphology, growth characteristics, and differentiation [1], [2]. Most normal endothelial cells require continuous signals from their environment to survive (mediated via adhesive interactions with other cells or extracellular matrix proteins) and loss of contact induces a specialized form of apoptosis, anoikis. The initiation and execution of anoikis is mediated by different pathways, all of which merge into the activation of caspases and downstream molecular pathways, culminating in the activation of endonucleases, DNA fragmentation and cell death [3]. As a result, failure to execute the anoikis program could result in adherent cells surviving under suspension conditions or proliferating at ectopic sites where the ECM proteins are different from the original ones. This deregulation in *anoikis* execution is emerging as a hallmark of cancer cells and contributes to the formation of metastasis in distant organs [4]. Indeed in neoplastic cells, alterations in cell-cell adhesion molecules, protein kinases or phosphatases, integrin-associated signalling molecules or apoptosis regulators can lead to resistance to the physiologically occurring anoikis, conferring by this way a constitutive pro-survival signal allowing dissemination of metastatic cancer cells [5]–[9].

For all steps in the metastatic cascade, the interaction of cells with the ECM is crucial [10]. Integrins are important mediators of cell adhesion to extracellular ligands and can transduce biochemical signals both into and out of cells [11], [12]. Vascular endothelial cells have been reported to express integrins α1β1, α2β1, α3β1, α5β1, α6β1, α6β4, αvβ3 and αvβ5 [11]. Integrins containing β1, β3 and β5 subunits interact with the microfilament system in focal adhesions [12]. Recent study provides evidence that integrin β5 facilitates cancer cell migration, anchorage-independent growth and tumor angiogenesis [13].

It is now becoming clear that additional transmembrane components can modify integrin-mediated adhesion. Syndecan-4 is a transmembrane heparan sulfate proteoglycan whose external glycosaminoglycan chains can bind extracellular matrix ligands and whose core protein cytoplasmic domain can signal during adhesion [14], [15]. The syndecans, including syndecan-1 and -4, selectively bind to various matrix components, growth factors and anticoagulant proteins through heparan sulfate glycosaminoglycan chains, and these interactions may facilitate important biological activities [16], [17]. Syndecan-1, -2, -4 and glypican-1 are expressed by vascular endothelial cells [18]–[20]. Endothelial cell line derived from rabbit aorta (EC) express mainly syndecan-4 [21]–[23].

Syndecan-4 is fundamental in cell adhesion and this adhesion plays important roles in the normal functions of cells, contributing to cellular organization and structure, proliferation and survival. This heparan sulfate proteoglycan is widely expressed but usually at low levels in normal tissue and unique among the syndecan family members to localize at sites of cell–matrix adhesions, specifically concentrated into focal adhesions together with integrins [24]–[27]. Its cytoplasmic domain can both bind to and potentiate the phospholipid-mediated activity of PKCα, which can itself be a focal adhesion component. Indeed, the insertion of syndecan-4 into focal adhesions requires PKC activity, suggesting that it may bind activated PKCα and both localize it to forming adhesions and potentiate its activity [28]–[31].

The expression of the syndecans can be altered under certain pathophysiological conditions, including the processes of tumor onset, progression and metastasis [32], [33]. Significant structural changes of heparan sulfate and overexpression of syndecan-4 were observed in the EJ-ras-transfected cells [21]. Upregulation of syndecan-4 has been noted in some carcinomas [34], [35] and such overexpression may correlate with increased tumor cell proliferation [36], [37]. So, alterations in the level of expression of the protein core, as well as heparan sulfate structure and/or density on heparan sulfate proteoglycans (HSPGs), can potentially make cancer cells highly versatile in modulating their behavior [38].

These and other results led us to investigate the role of syndecan-4 in the process of anoikis and cell transformation. We now report that anoikis-resistant endothelial cells display morphologic and molecular alterations, in particular, the overexpression of syndecan-4.

Our findings suggest that syndecan-4 may be involved in acquisition of resistance to detachment-induced cell death (anoikis resistance) in endothelial cells, thus contributing to cell transformation.

## Materials and Methods

### Reagents and Antibodies

Fibronectin (341635) and laminin (CC095) were acquired from Merck Millipore (Massachusetts, USA). Collagen type IV (354233) was obtained from BD Pharmingen (San Diego, CA, USA). Rabbit polyclonal antibody syndecan-4 (H-140) (sc-15350), rabbit polyclonal antibody HPA1 (H-80) (sc-25825) and mouse monoclonal antibody GAPDH (SC-66163) were purchased from Santa Cruz Biotechnology (Santa Cruz, CA, USA). Rabbit monoclonal antibody integrin β5 (D24A5) was purchased from Cell Signaling Technology, Inc. (Danvers, MA, USA). Alexa Fluor 488 phalloidin was obtained from Life Technologies (New York, USA). HRP-linked goat anti-mouse IgG (A4416) and HRP-linked goat anti-rabbit IgG (A6154) secondary antibodies were obtained from Sigma-Aldrich (St Louis, USA). Goat anti-rabbit IgG (H+L) highly cross-adsorbed labeled with Hilyte Fluor 594 (61056-05-H594) secondary antibody was obtained from Anaspec (Fremont, CA, USA).

### Cell Culture

Endothelial cell line derived from rabbit aorta (EC) [39], EC transfected with EJ-ras oncogene (EJ-ras EC) [21] and EC resistant to anoikis (Adh1^−^EC and Adh2^−^EC) were maintained in F12 medium (Life Technologies - New York, USA) supplemented with 10% fetal calf serum (FCS) (Athena, Campinas, SP, Brazil) at 37°C, 2.5% CO_2_. Serum-starved cultures were obtained by maintaining the cells in the presence of F12 containing 0.2% FCS, for 48 h at 37°C, 2.5% CO_2_ before stimulus with 10% FCS.

### Anchorage-Independent Growth Assays

Endothelial cells derived from rabbit aorta (EC) (10^5^ cells/ml) were plated on 1% agarose and cultured for 96 hours in F12 medium (Life Technologies - New York, United States) supplemented with 10% FCS (Campinas, Brazil) at 37°C, 2.5% CO_2_. Small spheroids were collected by decantation and plated on standard culture plates, favoring cell adhesion. Cells were allowed to proliferate to subconfluent growth. The deadhesion (spheroid formation) cycle was repeated for four or five times; after the last deadhesion step, spheroids were counted and plated by limiting dilution (0.5–1 spheroid/well) [40]. Two clones resistant to anoikis (Adh1^−^EC and Adh2^−^EC) were selected for more detailed studies.

### Cell Growth Analysis

Equal numbers (5×10^4^) of EC, EJ-ras EC, Adh1^−^EC and Adh2^−^EC cells were added into the different cell culture dishes and maintained in F12 medium (Life Technologies - New York, USA) supplemented with 10% fetal calf serum (FCS) (Athena, Campinas, SP, Brazil) at 37°C, 2.5% CO_2_. Every two days, cells were removed from the dishes, washed with PBS and, counted using a Neubauer chamber. Triplicate dishes were used for each time point (0, 2, 4, 6 and 8 days). Each experiment was repeated three times.

### Invasion Assay

The invasion assays was performed on 24-well plates using Polycarbonate Transwell of 8 µm pore size (Corning Inc. Lowell, MA, USA) coated with ECL cell attachment matrix (entactin, collagen IV and laminin) (1 mg/ml) (Merck Millipore, Massachusetts, USA). Briefly, the coated inserts were hydrated with warm F12 without serum in 2.5% CO_2_ at 37°C for two hours. A total of 5×10^4^ endothelial cells were seeded into the upper chamber containing F12 without serum, and the lower chamber contained F12 with 10% FCS. The cells were incubated at 37°C in 2.5% CO_2_ for 48 h. Non-invading cells were removed from the upper membrane by scrubbing with a cotton swab. Invading cells were fixed with 1% formaldehyde at room temperature for 30 minutes, stained with 1% toluidine blue (Sigma-Aldrich, St Louis, USA) dissolved in 1% sodium tetraborate for 15 minutes, and visualized under a light microscope (Carl Zeiss, Jena, Germany). The number of stained cells was counted in 10 microscope fields (20× magnification). Each experiment was repeated three times.

### Annexin V-FITC/PI Staining Assay

Apoptosis was assessed by the binding of annexin V conjugated with fluorescein isothiocyanate (FITC) to phosphotidylserine, which is externalized to the outer leaflet of the plasma membrane early during induction of apoptosis. Briefly, EC cells and its derived clones (1×10^6^) were resuspended in the binding buffer provided in the annexin V-FITC apoptosis detection kit II (BD Pharmingen, San Diego, CA, USA) and reacted with 5 µL of annexin V-FITC reagent and 5 µL of propidium iodide (PI) for 30 min at room temperature in the dark. Stained cells were analyzed by flow cytometry (FACSCalibur - Becton, Dickinson and Co, Franklin Lakes, NJ, USA), using the FlowJo program (Tree Star Inc., Ashland, OR, USA). Each experiment was repeated three times.

### BrdU Incorporation and Flow Cytometry for Cell Cycle

For BrdU incorporation, serum starved cultures were incubated with BrdU 10 µM in the absence or in the presence of 10% fetal calf serum (FCS) for 20 hours. BrdU uptake was determined essentially as recommended by the kit manufacturer (BrdU Cell Proliferation Assay - Merck Millipore – MA, USA). For cytometric DNA analysis, quiescent cultures were stimulated with 10% FCS for different periods of time. The cells were detached from the culture plate using trypsin and submitted to flow cytometric analysis as previously described [41] using the FlowJo software (Tree Star Inc., Ashland, OR, USA). Each experiment was repeated three times.

### Flow Cytometry for Surface Expression

Endothelial cells (1×10^6^ cells) were fixed with 2% paraformaldehyde and permeabilized with 0.01% saponin. The cells were then incubated for 2 h with the primary antibody (rabbit anti-syndecan-4 1: 300 and rabbit anti-integrin β5 1: 200). Afterwards, the cells were incubated for 1 h with goat anti-rabbit IgG-Hilyte Fluor 594-conjugated as a secondary antibody (1∶300). All antibodies were diluted in PBS with 1% BSA. Cells stained only with secondary antibody were used to subtract background fluorescence. Data analyses were performed on a BD FACSCalibur flow cytometer (BD Pharmingen, San Diego, CA, USA) using the WinMDI 2.9 software (J. Trotter, Scripps Research Institute, La Jolla, CA). The data represent the results of two different experiments.

### Adhesion Assays

Ninety six-well tissue culture plates (Corning/Costar – New York, USA) were submitted to attach to substrate (5, 10, 20 or 30 µg/ml of fibronectin, laminin or collagen IV) for 2 h at 37°C. The plates were washed with PBS and blocked with 1% BSA in PBS for 1 h at 37°C. Endothelial cells (5×10^4^ in F12-medium) were added to the plates and submitted to attach to substrate for 3 h at 37°C. At the end of incubation, the unattached cells were removed by washing the plates with PBS. Attached cells were fixed in methanol for 20 min, and stained with 0.8% crystal violet (Sigma-Aldrich – St Louis, USA) dissolved in 20% ethanol, and washed five times with PBS. The dye was then eluted with 50% ethanol 0.1 M sodium citrate, pH 4.2 and measured for absorbance at 540 nm using a microplate reader EZ Read 400 (Biochrom – MA, USA). For control, the non-adhesive substrate was prepared by coating the wells with 1% BSA for 60 min at 37°C. The experiments were performed in triplicates for each dose and repeated three times in different days.

### Western Blotting

Endothelial cells were lysed in buffer [50 mM Tris-HCl, pH 7.4, 1% Tween 20 and Halt Protease and Phosphatase Inhibitor Cocktail (Thermo Fisher Scientific Inc., Rockford, IL, USA)] for 2 h at 4°C. The homogenates were spun at 18,000 g for 5 min, and the supernatant was collected. Proteins were quantified using the BCA Protein Assay Kit (Thermo Fisher Scientific Inc., Rockford, IL, USA). Equal amounts of protein extracts (20 µg) were subjected to 10% SDS-PAGE and blotted onto a PVDF membrane (Merck Millipore, Massachusetts, USA). After blocking, the membrane was incubated with the primary antibody (rabbit anti-syndecan-4 1: 1000; rabbit anti-integrin β5 1: 1000, rabbit anti-HPA1 1: 1000 and mouse anti-GAPDH 1∶500) diluted in blocking buffer overnight at 4°C and followed by incubation with secondary antibodies (1∶10000) for 1 h at room temperature. The samples were detected by enhanced chemiluminescence using the SuperSignal West Pico Chemiluminescent Substrate (Thermo Fisher Scientific Inc., Rockford, IL, USA). An Alliance mini photodocumentation system from UVITEC (Cambrige, UK) was used to scan the films, and the UVIBAND MAX v1503b software (UVITEC - Cambrige, UK) was used to measure the amount of protein detected by each antibody. The experiments were performed in duplicates and repeated twice.

### Immunocytochemistry

Cells were cultured on 13.0 mm diameter glass coverslips (5×10^3^ cells/coverslip) in 24 well plates for 2 days followed by fixation with 2% formaldehyde in PBS (pH 7.4) for 20 min at room temperature. The cells were washed four times with PBS and once with PBS containing 0.1 M glycine. They were then permeabilized with PBS containing 1% BSA and 0.01% saponin for 15 min at room temperature. After this step, the cells were washed with PBS and incubated with rabbit anti-integrin β5 (1∶200) and Alexa Fluor 488 phalloidin (1∶250) diluted in PBS containing 0.01% saponin for 1 h at room temperature. After washing, the cells were incubated with goat anti-rabbit labeled with Hilyte Fluor 594 (1∶300) and DAPI (2 µg/ml) in PBS containing 0.01% saponin for 20 min at room temperature. The cells were washed and mounted in Fluoromount-G (2∶1) diluted in PBS and examined using an inverted confocal laser-scanning microscope (Leica TCS SP8, Leica Microsystems, Wetzlar, DE). Each figure shown in the results section corresponds to the best of two experiments.

### Extraction and Identification of Sulfated Glycosaminoglycans

Sulfated glycosaminoglycans synthesized by the cells were metabolically labeled with [^35^S]-sulfate (150 µCi/ml) in F-12 medium for 18 h at 37°C in 2.5% CO_2_ atmosphere. Afterwards, the culture medium was removed and the cells washed twice with F-12 medium and scrapped from the dish with 3.5 M urea in 25 mM Tris-HCl pH 7.8. Both to the medium and the cell extract 100 µg of carrier heparan sulfate, dermatan sulfate and chondroitin sulfate were added. The radioactive glycosaminoglycan free chains were prepared from the cell lysates and from the culture supernatants by incubation with 4 mg of maxatase (Biocon do Brasil Ltda, Rio de Janeiro, Brazil) for 4 h at 60°C. The [^35^S]-sulfated glycosaminoglycans were identified and quantified by agarose gel electrophoresis, as previously described in [42], [43]. The radioactive compounds were located by exposure of the gels (after fixation, drying and staining) to Cyclone storage phosphor screen (Packard Institute – Meriden, USA). For quantification, the radioactive bands were scrapped off the agarose gels, and counted in 5 ml of Ultima Gold (Packard) in a liquid scintillation spectrometer (TRI-CARB 2100TR, Packard). Protein was determined by the Coomassie blue assay. The experiments were performed in duplicates and repeated three times.

### RT-PCR

For RT-PCR, total RNA was isolated from the endothelial cells with the Trizol-reagent from Invitrogen (Carlsbad, CA, USA), and quantitated by spectrophotometer. RT-PCR was prepared using SuperScript One-Step RT-PCR with Platinum Taq (Life Technologies, New York, USA), according to the manufacturer’s instructions. All sequences available from the GenBankTM were included in the search for possible primer binding regions. Initially, the reverse transcription was performed at 45°C for 30 min. The following 5′ and 3′ primers were used in RT-PCR and the reaction performed for 40 cycles as specified for each pair of primers. Syndecan-4: Rev (5′-GAGTCGATTCGAGAG ACAGAG-3′); Fwd (5′-GTCCTTCTTCTTCATGCGGTAC-3′), denaturation at 94°C for 1 min, annealing at 55°C for 1 min, extension at 72°C for 2 min). The amplified product is 459 bp in size. GAPDH: Rev (5′-ATGCCATCACTGCCACCCAG-3′); Fwd (5′-CATGCCAGTGAGCTTCCCGTT-3′), denaturation at 94°C for 1 min, annealing at 55°C for 1 min, extension at 72°C for 1 min. The amplified product is 158 bp in size. Primers were synthesized by GENSET Corporation (San Diego, USA). After cycling, each sample was fractionated on 1% agarose gel in Tris-acetate buffer, stained with ethidium bromide, and visualized by UV fluorescence. The sizes of RT-PCR products were determined using the 100 bp DNA ladder (Life Technologies, New York, USA). The products were digitalized using Alliance 4.7 Video Documentation System (Uvitec Cambridge – Cambridge, UK), and quantitative analysis performed with UviBand Software (Uvitec Cambridge – Cambridge, UK). The experiment was repeated four times.

### Quantitative Real-Time PCR (qPCR)

For quantitative PCR (qPCR), 10 µg of total RNA per sample was reverse transcribed to cDNA with anmiScript Reverse Transcription Kit (Qiagen, Hilden, DE) and then 50 ng of cDNA was analyzed using the miScript SYBR Green Kit (Qiagen, Hilden, DE) with a ABI 7500 real-time PCR instrument (Applied Biosystems, Foster, CA, USA), according to the manufacturer’s instruction, respectively. The primers are used as follow: syndecan-4 Fwd (5′-AGCCTGGGCAGGTCGTGTCT-3′) and syndecan-4 Rev (5′-GTGGTTCCCCTCACCGCAGC-3′); heparanase Fwd (5′-CAAGAAGGTTGGGTTCTGAGGAGA-3′) and heparanase Rev (5′-GCATCGAAGGTG AGCAAGGATGG-3′); GAPDH Fwd (5′-CGCTTCGCTCTCTGCTCCTCC-3′) and GAPDH Rev (5′- TGGTGACCAGGCGCCCAATAC-3′). PCR was performed at 95°C for 15 s and 60°C for 60 s for 40 cycles. GAPDH was used as an internal standard to normalize mRNA levels for differences in sample concentration and loading. Fold changes in the expression of each target mRNA relative to GAPDH was calculated based on the threshold cycle (Ct) as 2−Δ (ΔCt), where ΔCt = Ct target- Ct GAPDH and Δ (ΔCt) = ΔCtAdh-EC−ΔCtEC. The post-amplification melting curve analysis was performed to confirm whether the nonspecific amplification was generated from primer-dimers. Quantitative PCR reactions were performed in triplicate. Results were derived from a total of four independent experiments.

### Statistical Analysis

All data are expressed as the mean ± standard error of the mean. Statistical significance was assessed using analysis of Student's t-test. P values <0.05 were considered statistically significant.

## Results

### Characterization of Anoikis-Resistant Endothelial Cells

Endothelial cells were submitted to stressful conditions by blocking adhesion to substrate, as described in methods. As expected for a nontumorigenic immortalized cell line, the great majority of these cells underwent apoptosis induced by adhesion blockade (anoikis). After cultivating endothelial cells in agarose-coated plates for 96 hours, few spheroids were observed. Anoikis-resistant EC cells were cultured in adherent conditions and submitted to new deadhesion cycles. Distinct lineages (Adh1^−^EC, Adh2^−^EC, Adh3^−^EC, Adh4^−^EC, Adh5^−^EC and others) were obtained by limiting dilution after four deadhesion cycle of EC cells (1 spheroid/well) and two clones (Adh1^−^EC and Adh2^−^EC) were then selected for more detailed studies.

**Figure 1:**
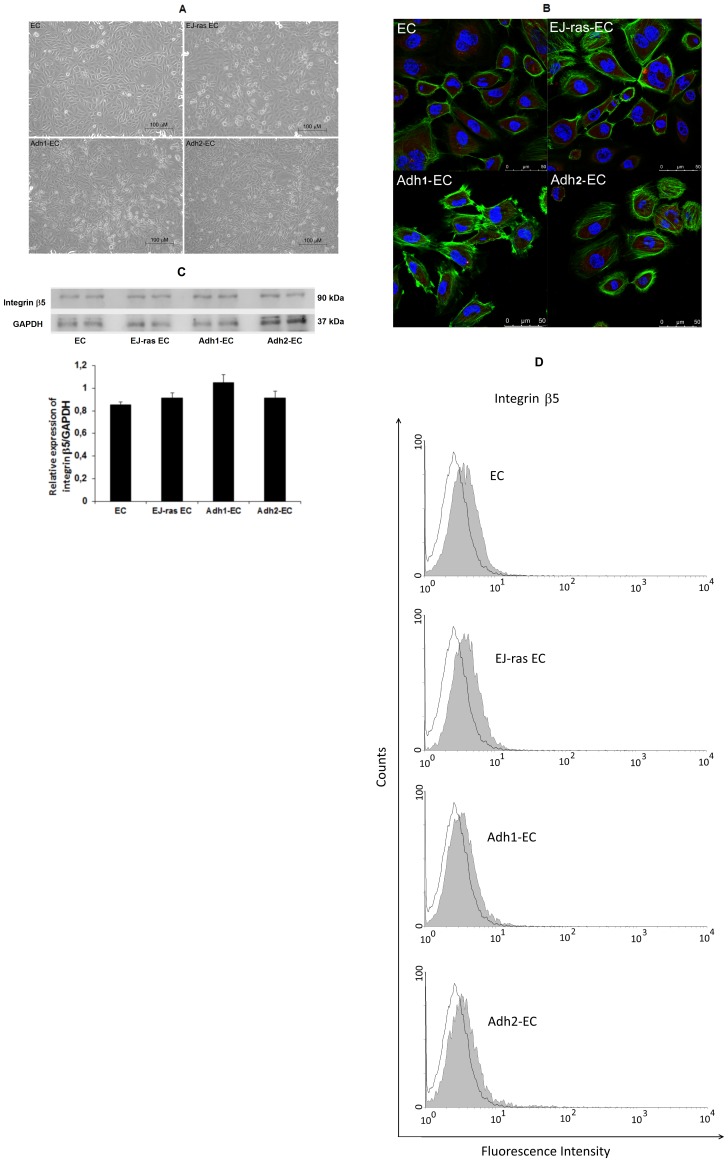
Morphologic characteristics and integrin β5 expression of EC-derived cell lines. (A) Parental (EC), EJ-*ras* transfected endothelial cells (EJ-ras EC), and anoikis-resistant endothelial cells (Adh1^−^EC and Adh2^−^EC) were maintained in culture for 7 days and photographed under a Nikon phase contrast microscope. Bar = 100 µm. (B) Immunofluorescent analysis of F-actin and integrin β5 in EC, EJ-ras EC, Adh1^−^EC and Adh2^−^EC cells. Actin filaments were stained with Alexa Fluor-488 phalloidin (green) and integrin β5 was stained with anti-integrin β5 antibody followed by secondary Hilyte Fluor-594-labelled antibody (red). Nucleus stained with DAPI (blue). Scale bar: 50 µm. (C) The protein expression levels of integrin β5 were assessed by western blot analysis. GAPDH is shown as a protein loading control. Histogram depicting integrin β5 protein levels normalized to GAPDH. (D) Flow cytometry analysis for cell surface presentation of integrin β5 in EC, EJ-ras EC, Adh1^−^EC and Adh2^−^EC cells. Filled histogram, cells treated with anti-integrin β5 and secondary antibody; opened histogram, cells treated only with secondary antibody. In B, C and D, two independent experiments were performed in duplicates. The bars represent the standard error.

**Figure 2:**
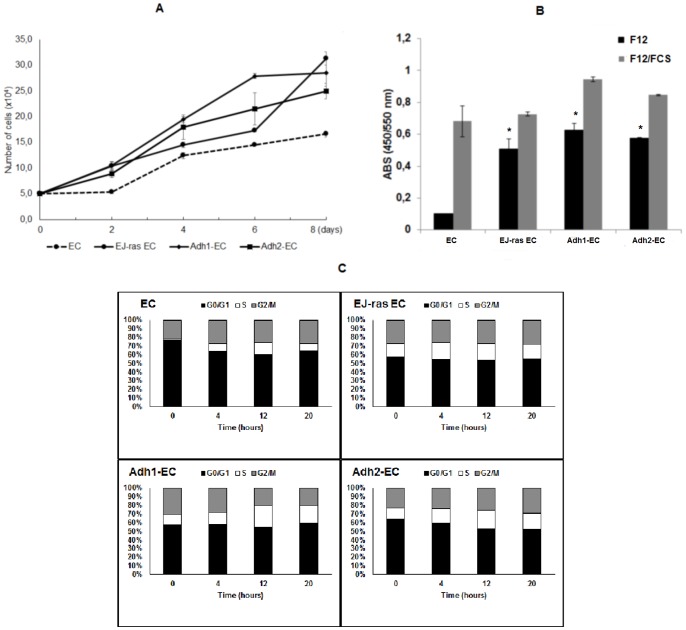
Proliferation and cell cycle of EC-derived cell lines. (**A**) Growth curves of EC, EJ-*ras* transfected cells (EJ-ras EC), and EC anoikis resistant (Adh1^−^EC and Adh2^−^EC). (B) BrdU incorporation for 20 h. Serum-starved EC, EJ-ras-transfected cells (EJ-ras EC) and EC anoikis resistant (Adh1^−^EC and Adh2^−^EC) were serum-starved, as described in Methods, and stimulated to proliferate by the addition of 10% FCS. (C) Cell cycle distribution of EC, EJ-*ras* transfected cells (EJ-ras EC), and EC anoikis resistant (Adh1^−^EC and Adh2^−^EC). Histograms show the proportion of cells at different stages in the cell cycle (DNA content of propidium iodide–stained nuclei) analyzed by flow cytometry. ABS: absorbance. All experiments were repeated three times. The bars represent the standard error. * P≤0.05.

**Figure 3:**
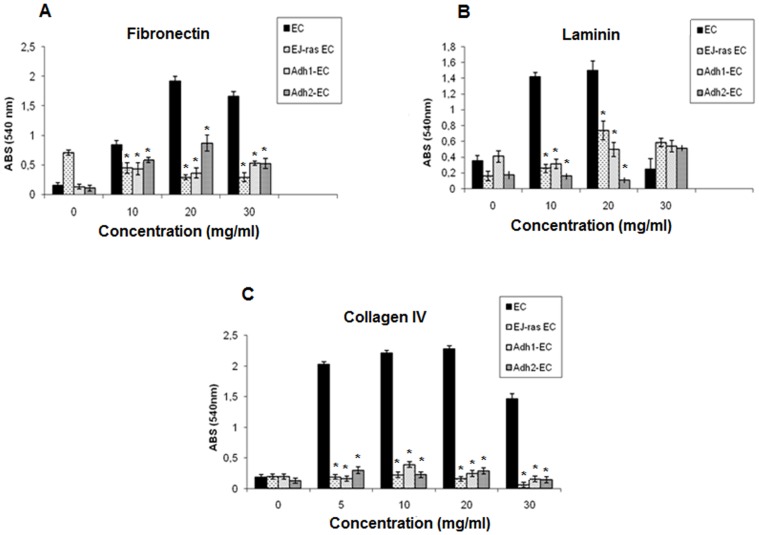
Adhesion rate of EC-derived cell lines. (A), (B) and (C) Assay adhesion of parental (EC), EJ-ras transfected endothelial cells (EJ-ras EC), and anoikis-resistant endothelial cells (Adh1^−^EC and Adh2^−^EC) on fibronectin, laminin, and collagen IV, respectively. ABS: absorbance. The experiments were performed in triplicates and repeated three times. The bars represent the standard error. * P≤0.05.

The selected clones were analyzed with respect to cell morphology, cell growth and adhesiveness. EC (not tumorigenic) and EJ-ras EC (tumorigenic) were used as controls. All cell lines were maintained in culture and observed under a phase contrast microscope (Fig. 1A). Altered morphology was observed in all EC–derived cell lines, which demonstrated stable phenotypic characteristics. Cell lines obtained after submitting EC cells to adhesion impediment cycles are arranged in several layers of cells, did not show density inhibition or growth arrest upon serum starvation, as described for EJ-ras EC cells [21]. In contrast, the parental line display in a cell monolayer, showing density inhibition. Recent study have implicated integrin β5 in tumor growth in anchorage-independent condition [13]. To investigate the role of integrin β5 in anoikis resistance, we analyzed the expression of integrin β5 in all cell lines. Actin filaments and integrin β5 were analyzed by confocal fluorescence microscopy (Fig. 1B). Anoikis-resistant endothelial cells showed irregular shape, variable disorganization and increase in the number of actin filaments. No changes in the cellular distribution of integrin β5 were observed. Western blot analysis showed that protein expression levels of integrin β5 were similar in all cell lines (Fig. 1C). These data were corroborated by flow cytometric analysis (Fig. 1D). In addition, the cell growth rate nearly doubled for EJ-ras EC, Adh1^−^EC and Adh2^−^EC cells in relation to EC cells (Fig. 2A). The cell lines obtained after four deadhesion steps showed lower adhesiveness to fibronectin, laminin and collagen IV in relation to EC cells. Similar results were observed in EJ-ras EC cells (Figs. 3A, B and C).

### BrdU Incorporation and Flow Cytometry of Anoikis-Resistant Endothelial Cells

Serum-starved EC, EJ-ras EC, Adh1^−^EC and Adh2^−^EC cells were labeled with BrdU 10 µM in the absence or in the presence of 10% FCS for 20 hours. Adh1^−^EC, Adh2^−^EC and EJ-ras EC cells incorporate BrdU practically to the same extent, both in the presence and in the absence of FCS, as opposed to the parental cells, which are highly dependent on serum for BrdU incorporation (Fig. 2B).

The results obtained with flow cytometry confirm these observations. Serum-starved EC, EJ-ras EC, Adh1^−^EC and Adh2^−^EC cells cycle was analyzed by flow cytometry based on their DNA content. EC and EC–derived cell lines were stimulated to enter the S phase by FCS addition. However, the growth response to FCS exhibited by the parental endothelial cells is not present in the Adh1^−^EC, Adh2^−^EC and EJ-ras EC cells (Table 1, Fig. 2C). Anoikis-resistant endothelial cells are not synchronized by serum starvation, as described for EJ-ras EC cells [21].

**Table 1:** Cell cycle of EC-derived cell lines.

	EC (%)			EJ-ras EC (%)			Adh1^−^EC (%)			Adh2^−^EC (%)		
Time (h)^a^	G_0_/G_1_	S	G_2_/M	G_0_/G_1_	S	G_2_/M	G_0_/G_1_	S	G_2_/M	G_0_/G_1_	S	G_2_/M
**0**	76,7	2,5	20,8	57,7	14,9	27,4	57,7	11,7	30,6	64,4	12,5	23,1
**4**	63,8	9,2	27,0	54,6	19,9	25,5	57,8	13,9	28,3	59,5	16,8	23,7
**12**	60,2	14,2	25,6	53,8	19,0	27,3	54,9	24,7	20,4	53,1	21,2	25,7
**20**	64,5	8,2	27,3	55,4	17,1	27,5	59,4	20,1	20,5	52,8	17,9	29,3

a Cells were kept in 0.2% FCS for 48 h, and stimulated with 10% FCS. The cells were harvested for flow cytometric analysis of DNA content at various time periods after stimulation. The numbers represent an average of three different experiments with an error of 5%.

### Invasion Capacity and Percentage of Apoptosis in Anoikis-Resistant Endothelial Cells

**Figure 4:**
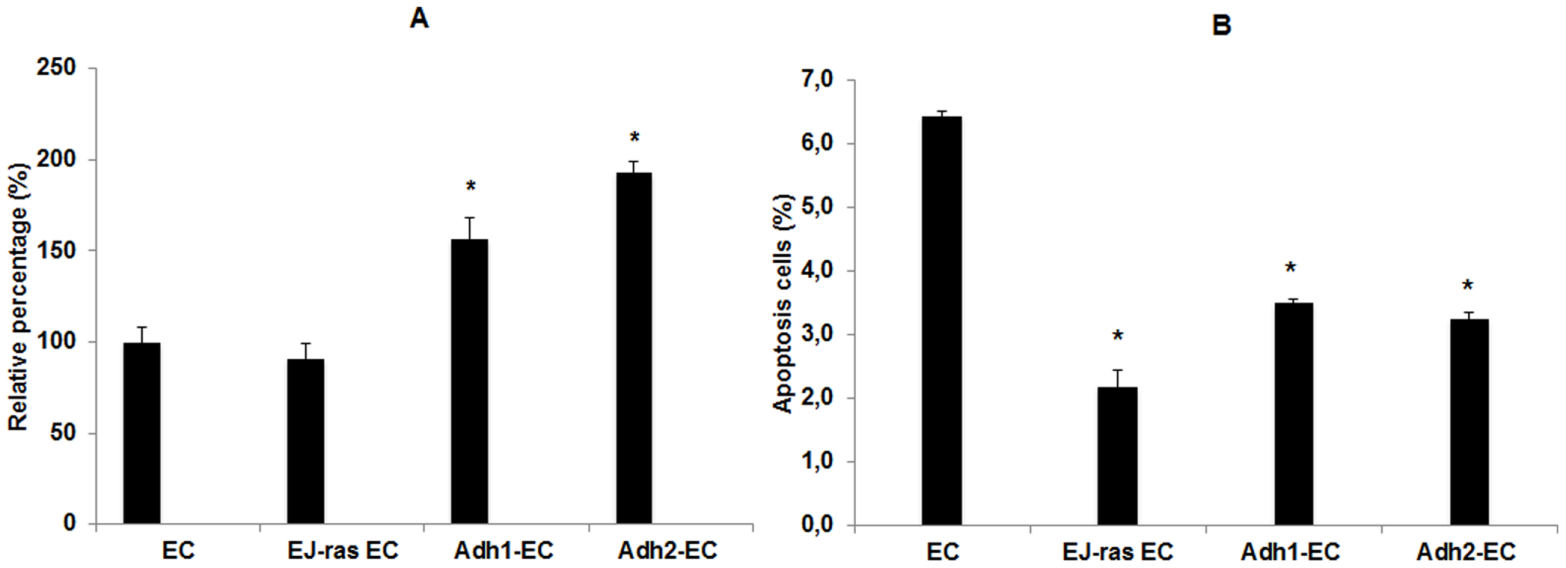
Invasion capacity and percentage of apoptosis in EC-derived cell lines. (A) Values of relative invasiveness of EJ-ras EC, Adh1^−^EC and Adh2^−^EC cells normalized with those of EC cells (100%). Invasion activity of EC-derived cell lines was analyzed by using a Transwell coated with ECL cell attachment matrix as described in Methods. (B) Percentage of apoptotic cells detected by Annexin V-FITIC/PI double staining method in EC, EJ-*ras* transfected cells (EJ-ras EC), and EC anoikis resistant (Adh1vEC and Adh2^−^EC) cells. All experiments were repeated three times. The bars represent the standard error. * P≤0.05.

As anoikis resistance is a key factor in tumor metastasis, we decided to analyze the invasiveness and apoptosis rates in anoikis-resistant endothelial cells. To analyze the invasiveness, EC, EJ-ras EC, Adh1^−^EC and Adh2^−^EC cells were added into the plates using polycarbonate transwell coated with ECL cell attachment matrix (Merck Millipore). As shown in Fig. 4A, invasion chamber analysis demonstrated that, compared to EC cells (100%), the invasion rate of Adh1^−^EC, Adh2^−^EC and EJ-ras EC cells were 156%, 193% and 91%, respectively. To determine the percentage of apoptotic cells, EC, EJ-ras EC, Adh1^−^EC and Adh2^−^EC cells were harvested and stained with Annexin V-FITC and propidium iodide (PI) (BD Pharmingen). Stained cells were analyzed by flow cytometry. The results obtained with flow cytometry revealed a significant decrease in apoptosis in Adh1^−^EC, Adh2^−^EC and EJ-ras EC cells (3.5%, 3.2% and 2.2%, respectively) compared with EC cells (6.4%) (Fig. 4B). The results indicate that anoikis-resistant endothelial cells exhibit characteristics of tumorigenic cells as high invasive potential and low rate of apoptosis.

### Heparan Sulfate and Chondroitin Sulfate Expression Is Increased in Anoikis-Resistant Endothelial Cells

**Figure 5:**
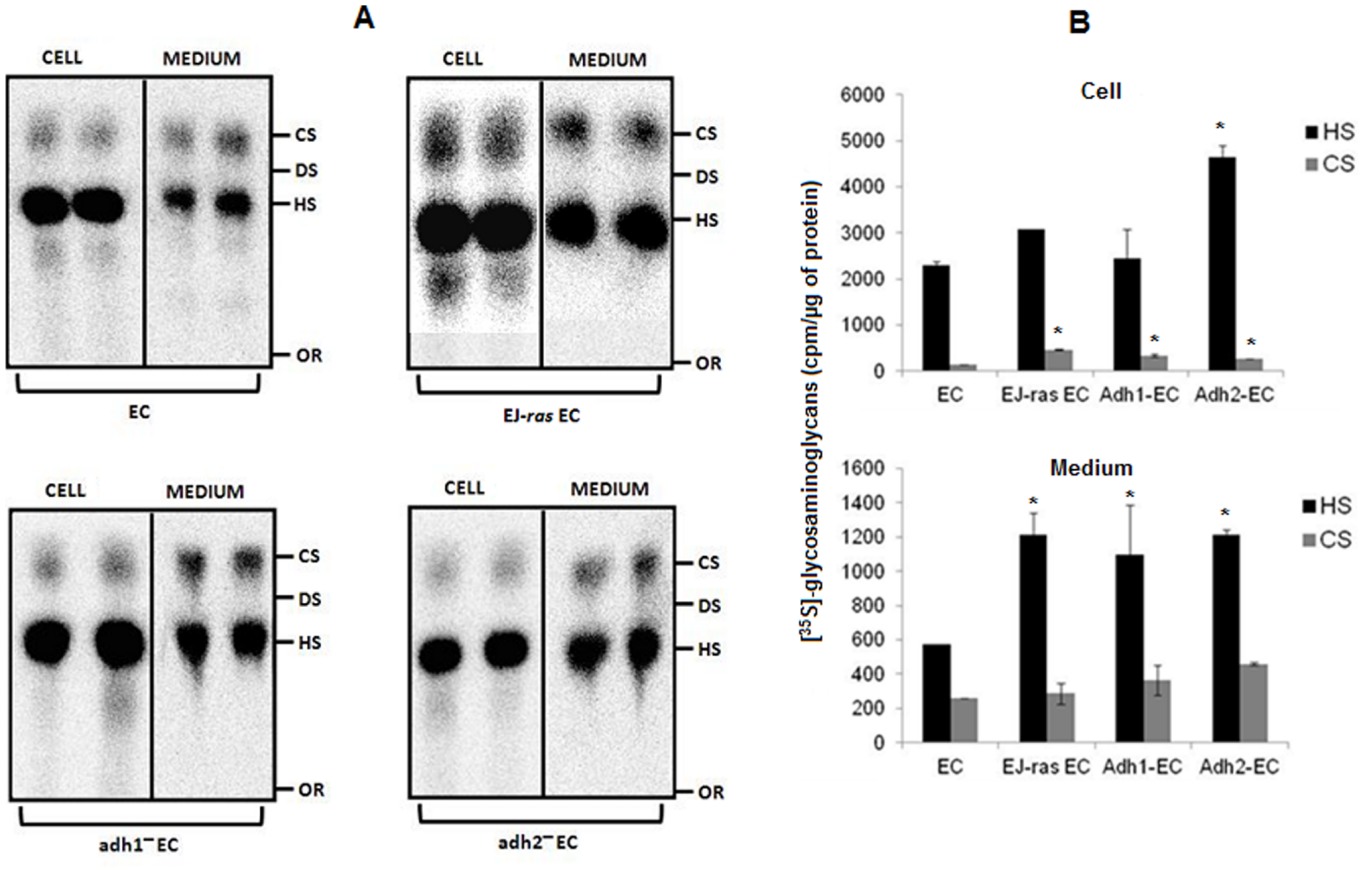
[^35^S] sulfated glycosaminoglycans synthesized by EC, EJ-ras EC, Adh1^−^EC and Adh2^−^EC cells. (A) Endothelial cells were exposed to [^35^S]sulfate for 18 h. The radioactive glycosaminoglycan-free chains were prepared, from both cells and conditioned medium, by incubation with proteolytic enzyme. Aliquots were subjected to electrophoresis for 60 min in 0.6% agarose (0.05 M 1,3-diaminopropane-acetate buffer pH 9.0) [38], [39]. The radioactive compounds were located in the gels, as described in Methods. CS: chondroitin sulfate; DS: dermatan sulfate; HS: heparan sulfate; OR: origin. (B) Quantification of the experiment shown in A. For further details, see Methods. The experiments were performed in duplicates and repeated three times. EC: parental endothelial cells; EJ-ras EC: EJ-ras transfected endothelial cells; Adh1^−^EC and Adh2^−^EC: anoikis-resistant endothelial cells. The bars represent the standard error. * P≤0.05.

Endothelial cells were exposed to [^35^S]sulfate for 18 h and aliquots of the cell extracts and of the culture medium were treated with protease and subjected to electrophoresis, as described in Methods. Fig. 5A shows an increase in the expression of heparan sulfate and chondroitin sulfate present in the Adh-EC and EJ-ras EC cells, when compared to the parental line. An increase in the amount of heparan sulfate and chondroitin sulfate secreted to the medium was also observed. The quantitative data of these experiments (Fig. 5B) indicate that significant differences are found in the levels of chondroitin sulfate present in the cells and in the levels of heparan sulfate secreted to the medium by parental, Adh-EC and EJ-ras EC cells. These data led us to investigate the levels of syndecan-4 and heparanase in endothelial cells.

### Syndecan-4 and Heparanase Expression in Anoikis-Resistant Endothelial Cells

**Figure 6:**
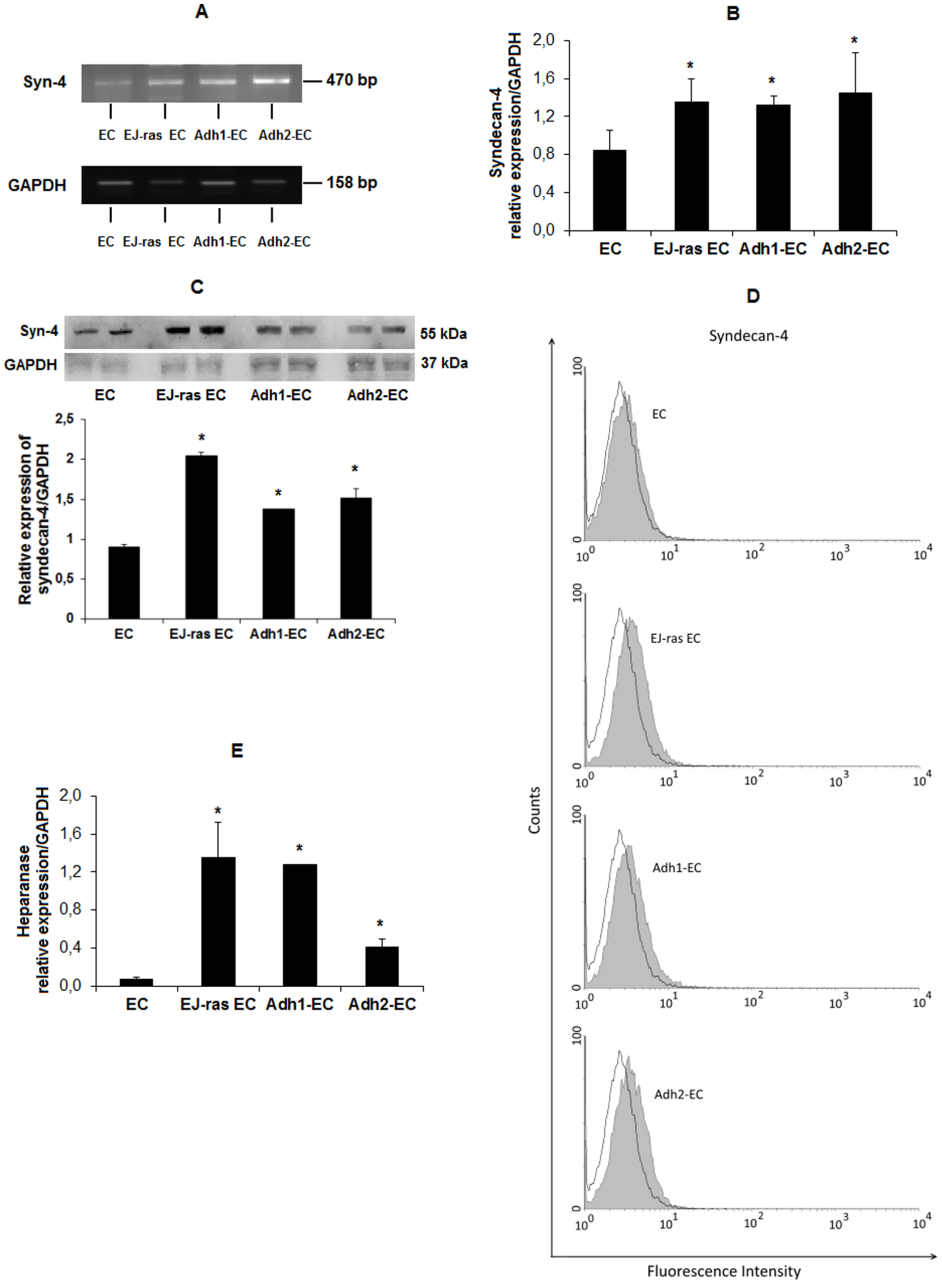
Expression of syndecan-4 and heparanase in EC, EJ-ras EC, Adh1-EC and Adh2-EC cells. (A) Representative RT-PCR products fractionated in 1% agarose gel electrophoresis and stained with ethidium bromide, showing bands of the expected sizes. (B) Expression of syndecan-4 detected by RT-qPCR. GAPDH was used as a loading control. (C) The protein expression levels of syndecan-4 were assessed by western blot analysis. GAPDH is shown as a protein loading control. Histogram depicting syndecan-4 protein levels normalized to GAPDH. (D) Flow cytometry analysis for cell surface presentation of syndecan-4 in EC, EJ-ras EC, Adh1-EC and Adh2-EC cells. Filled histogram, cells treated with anti-syndecan-4 and secondary antibody; opened histogram, cells treated only with secondary antibody. (E) Expression of heparanase detected by RT-qPCR. GAPDH was used as a loading control. In A, B and E, the experiments were repeated four times. In C and D two independent experiments were performed in duplicates. EC: parental endothelial cells; EJ-ras EC: EJ-ras transfected endothelial cells; Adh1-EC and Adh2-EC: anoikis-resistant endothelial cells. The bars represent the standard error. * P≤0.05.

In order to examine the gene expression levels of syndecan-4 (syn-4) and heparanase (HPSE) by parental, Adh-EC and EJ-ras EC cells, we used RT-PCR and qPCR analyses. It is clear that anoikis-resistant endothelial cells show higher levels of expression of syndecan-4 (Fig. 6A). The relative values of expression of syndecan-4 normalized with respect to GAPDH levels show a 1.55, 1.71 and 1.59-fold increase for the Adh1-EC, Adh2-EC and EJ-ras EC cells, respectively, when compared to the parental cells (Fig. 6B). As shown in Fig. 6E, the qPCR demonstrated a statistically significant difference in the expression of heparanase in the parental, Adh-EC and EJ-ras EC cells. Heparanase levels were increased 16.4, 5.3 and 17.3-fold in Adh1-EC, Adh2-EC and EJ-ras EC cells, respectively, when compared to the parental cells. In addition, we examined the protein expression levels of syndecan-4 in all cell lines using western blot (Fig. 6C) and flow cytometry (Fig. 6D) techniques and confirmed that EC–derived cell lines express more syndecan-4 than parental cells. S1 Fig. shows the ratio of active HPSE:pro-enzyme HPSE (active HPSE:pro-enzyme HPSE/GAPDH) determined by Western Blot analysis. It was demonstrated that Adh1-EC and Adh2-EC cells have an increase of active HPSE (50 kDa), respectively, 11% and 21%, while EJ-ras EC cells present an increase of 26%, compared to EC cells. These combined results indicated that EJ-ras EC cells, as well as, both anoikis-resistant endothelial cells (Adh1-EC and Adh2-EC), present higher levels of active HPSE comparing to EC cells. Interestingly, increased ratio of active HPSE:pro-enzyme HPSE in different cell lineages (S1 Fig.), follows the same profile of protein levels of syndecan-4 (Fig. 6C).

## Discussion

In the absence of attachment to ECM or upon cell adhesion to inappropriate location, cells undergo a particular type of apoptosis, termed anoikis. Anoikis has been described in several cell types, although it appears that cells of different tissue origin activate dissimilar pathways leading to anoikis. The physiological relevance of anoikis is confirmed by the fact that cancer cells lines, rather than normal epithelial cells, are usually not sensitive to anoikis, and many have developed anchorage independence, meaning they do not require adhesion to ECM to proliferate and survive [44], [45]. Here, we show that repetitive adhesion blockade events can lead to endothelial cell transformation. Anoikis-resistant endothelial cells (Adh-EC) display morphological alterations, high rate of proliferation, poor adhesion to fibronectin, laminin and collagen IV and deregulation of the cell cycle, becoming less serum-dependent, as shown by the ratios of BrdU incorporation and flow cytometry in the absence and in the presence of FCS, in contrast with the parental cells. Furthermore, EC–derived cell lines display a high invasive potential and a low rate of apoptosis. This is accompanied by an increase in the levels of heparan sulfate and chondroitin sulfate as well as by changes in the expression of syndecan-4 and heparanase. Similar results were observed in endothelial cells transfected with EJ-ras oncogene (EJ-ras EC) [21].

Previous studies have shown that repetitive adhesion blockade events are sufficient to induce malignant transformation in nontumorigenic immortalized melanocytes [40]. Multiple factors with frequently overlapping functions have been reported to confer anoikis suppression to tumor cells by stimulating survival pathways. One, however, could propose that anoikis resistance not only confers the cell with the ability to survive without attachment to the ECM but may also provide a molecular signature that secures their successful migration, invasion, and metastasis to distant sites [46]. Endothelial cells are protected by anoikis when they are adherent on permissive ECM proteins.

Tumor progression resulting in cancer metastasis is a multistep process, which is dependent on dynamic changes in adhesive and migratory ability of tumor cells. Integrins are the major cell surface receptors mediating cell-to-extracellular matrix adhesion and are involved in the regulation of focal adhesion turnover and survival signaling, which are key events in the process of tumor growth and tumor cell migration [47]. Cooperatively with the integrins, cell adhesion is also organized by cell surface heparan sulfate proteoglycans, syndecans [48]. Of all 4 syndecan genes, syndecan-4 (SDC4) is the only ubiquitously expressed member and functions as an integrin co-receptor in cell adhesion–promoting mitogen-activated protein kinase signaling pathways [48], [49].

Many endothelial cells express HSPGs at their cell surface, which include syndecans and glypicans [18]–[20]. Endothelial cells derived from rabbit aorta (EC) express predominantly syndecan-4. HS is the main glycosaminoglycan synthesized by these cells [21]–[23]. Acquisition of anoikis resistance leads to an increase in the amount of HS and syndecan-4 synthesized by endothelial cells. Experimental evidences suggest that heparan sulfate proteoglycan (HSPG) play a role in cell spreading, cellular recognition, cellular adhesion and growth control [50]–[58]. In addition, several reports describe high affinity association of heparin-like molecules with growth factors [59]–[62], implying that heparan sulfate effects on cell growth are likely to be mediated by growth factors [61]–[69]. Syndecan-4 mediates breast cancer cell adhesion and spreading [17] but also binds proangiogenic growth factors and cytokines and modulates growth factor/growth factor receptor interactions regulating angiogenic processes [70], [71].

Several studies have correlated the overexpression of syndecan-4 with increased tumor cell proliferation [36], [37], [72]. Up-regulation of syndecan-4 is associated with the development and metastasis of renal cell carcinoma, possibly by increasing the cell migratory potential and survival through integrin-mediated signaling [73]. Up-regulation of syndecan-4 has also been noted in hepatocellular carcinomas and malignant mesotheliomas [34], [35]. Significant structural changes of heparan sulfate and overexpression of syndecan-4 were observed in the EJ-ras-transfected cells [21]. HS chains bind a multitude of proteins and ensure that a wide variety of bioactive molecules (e.g., heparin-binding growth factors, chemokines, lipoproteins, and enzymes) cling to the cell surface and ECM. HSPGs can thus influence a variety of normal and pathologic processes, among which are tissue repair, neurite outgrowth, inflammation and autoimmunity, tumor growth and metastasis, vasculogenesis and angiogenesis [26], [74]–[77].

Because of the important and multifaceted roles of HSPGs in cell physiology, their cleavage is likely to alter the integrity and functional state of tissues and to provide a mechanism by which cells can respond rapidly to changes in the extracellular environment. Enzymatic degradation of HS is, therefore, likely to be involved in fundamental biological phenomena, ranging from pregnancy, morphogenesis, and development to inflammation, angiogenesis, and cancer metastasis [78]. Heparanase is an endo-β-glucuronidase that is capable of degrading heparan sulfate chains of proteoglycans, a key component of the extracellular matrix and the basement membrane. The oligosaccharides so generated lead to the release of a variety of bioactive molecules, such as growth factors, chemotactic agents, and angiogenic agents, which are then deposited in the extracellular matrix and basement membrane. These molecules can stimulate cell proliferation, increase cell survival, and promote angiogenesis, morphogenesis, and vascularization [79], [80].

The expression of heparanase was investigated in EC–derived cell lines. Anoikis-resistant endothelial cells show an increase in the expression of heparanase. Most studies investigating heparanase have focused on its regulated expression at different stages of cancer progression, and its overexpression in tumor cells has also been reported to correlate with metastatic potential and poorer prognosis [81], [82]. Heparanase and glycosaminoglycans can modulate initial events of renal cell carcinoma development [83]. In bone tissue, heparanase-1 overexpression creates a complex phenotype that typically results in osteogenesis and increased bone mass [84].

The combined results provided evidences that increased syndecan-4 expression and shedding may be modulated by increased expression of active heparanase (50 kDa) in Adh1-EC and Adh2-EC cells. It was also described in the literature the correlation between heparanase, metaloproteases and syndecan expression or shedding [85], [86].

In conclusion, this study revealed that the acquisition of anoikis resistance induced syndecan-4 up-regulation in endothelial cells. Acquisition of resistance to anoikis is a potentially crucial step in endothelial cell transformation. A better understanding of the mechanisms underlying anoikis resistance may provide insight into the biology of cancer and identify novel therapeutic targets for prevention of metastasis formation.

## Supporting Information

S1 Fig Expression of heparanase protein. The protein expression levels of heparanase (HPSE) were assessed by western blot analysis. GAPDH is shown as a protein loading control. Histogram depicting the ratio of active HPSE:pro-enzyme HPSE (active HPSE:pro-enzyme HPSE/GAPDH). The experiment was performed in duplicate and repeated twice. Active HPSE: 50 kDa; Pro-enzyme HPSE: 65 kDa. EC: parental endothelial cells; EJ-ras EC: EJ-ras transfected endothelial cells; Adh1-EC and Adh2-EC: anoikis-resistant endothelial cells.
(TIF)
