# Supplementary figures and images for: Acquisition of Anoikis Resistance Up-Regulates Syndecan-4 Expression in Endothelial Cells
Source: PLoS One. 2014 Dec 30;9(12):e116001. doi: 10.1371/journal.pone.0116001 (PMC4280138; doi:10.1371/journal.pone.0116001)

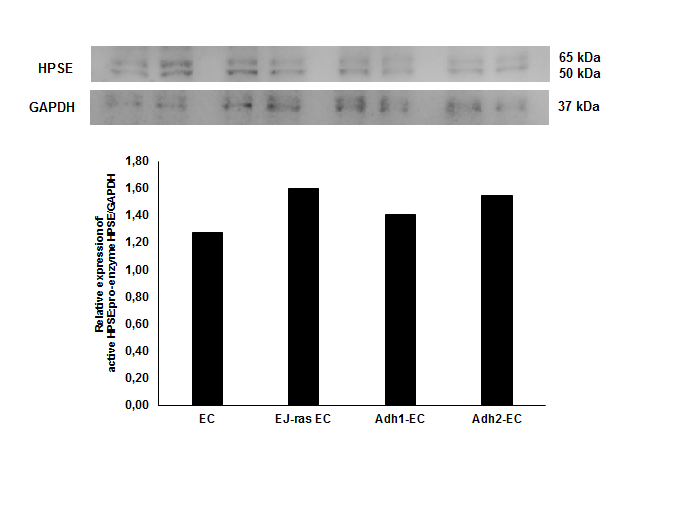

Supplement: S1 Fig — Expression of heparanase protein. The protein expression levels of heparanase (HPSE) were assessed by western blot analysis. GAPDH is shown as a protein loading control. Histogram depicting the ratio of active HPSE:pro-enzyme HPSE (active HPSE:pro-enzyme HPSE/GAPDH). The experiment was performed in duplicate and repeated twice. Active HPSE: 50 kDa; Pro-enzyme HPSE: 65 kDa. EC: parental endothelial cells; EJ-ras EC: EJ-ras transfected endothelial cells; Adh1-EC and Adh2-EC: anoikis-resistant endothelial cells. (TIF) [file pone.0116001.s001.tif]
